# Genomics Helps Explain Why Some Like It Hot

**DOI:** 10.1371/journal.pbio.0030317

**Published:** 2005-08-23

As warm-blooded creatures, humans and other mammals maintain a relatively stable body temperature that buckles under the stress of intense heat (or cold). When the heat gets too high, we develop fevers and weaken, and our proteins destabilize and degrade—in some cases, such reactions can prove fatal. But some organisms appear to defy nature (as we think of it) by flourishing in extremely high temperatures. The archaeal microbe Pyrobaculum aerophilum, for example—originally found in a boiling marine water hole in Italy—thrives at ∼100 °C (212 °F). Similarly, the bacterium Thermus thermophilus grows at temperatures between 48 °C and 85 °C (118–185 °F).

Such organisms are of interest for many reasons—not least of which is to understand the mechanisms that engineer their heat resistance, or thermostability. How do these thermophilic bacteria and archaea manage to maintain active, stable proteins at such high temperatures? In an elegant demonstration of how the ever-growing publicly available genome sequence and protein structure data can be analyzed, Todd Yeates and colleagues identify one answer to this question.

The authors found that proteins from P. aerophilum and T. thermophilus, along with some other thermophiles, have many disulfides, which are known to improve stability. Disulfides are covalent bonds that form when the sulfhydryl groups (a sulfur and a hydrogen atom) of two spatially proximate cysteines (one of the 20 amino acid building blocks of proteins) are oxidized. When conditions are right, the two hydrogen atoms are removed by other molecules in the cell dedicated to that purpose, and the remaining sulfur atoms form a bond. [Fig pbio-0030317-g001]

**Figure pbio-0030317-g001:**
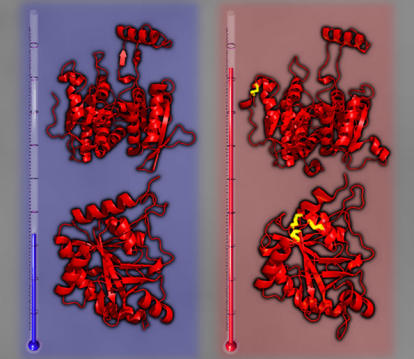
Some thermophilic bacteria can thrive in extreme heat because their proteins have an abundance of disulfides (yellow, above), covalent bonds between sulfur atoms that improve stability and likely boost heat-tolerance

The authors mapped sequences of intracellular genes from 199 prokaryote genomes onto sequence-related proteins with known three-dimensional structures. The resulting structural models reveal when disulfide bonds are likely to form. A pronounced bias was found for disulfides in a set of thermophilic genomes. To prove that these predictions really do form disulfide bonds, the authors solved the structure of one protein from P. aerophilum—which was indeed stabilized by three disulfide bonds.

Disulfide bonds form more commonly outside or between cells in multicellular organisms, where the environment is ideal for two cysteines to cozy up and bond in an oxidative extracellular location. The high numbers of bonds observed in these single-cell prokaryotes not only help explain thermostability but also challenge our ideas of how disulfide bonds form. Given the presumed difficulty for disulfides to form in such organisms, the authors set out to look for any proteins that might help explain the mystery. They investigated which proteins are present in the disulfide-rich organisms as compared with the proteins in other organisms (also known as phylogenetic profiling). The authors discovered that all of the disulfide-rich thermophiles had something else in common: they all encode a protein not seen in other organisms, called protein disulfide oxidoreductase (PDO). As its name suggests, this protein likely plays a key role in the formation of disulfides in these heat-tolerant bugs.

Yeates and colleagues have considerably advanced our understanding of how proteins withstand and remain functional at high temperatures in these thermophilic organisms (via additional stabilizing disulfide bonds). Yet, since this correlation of extra disulfides and the PDO is not common to all thermophiles, it seems likely that this is not the only method employed in heat resistance. Probably a finely tuned concert of different mechanisms works in synchrony to enable thermophiles to flourish in extreme conditions. As the authors show here, it's likely that genome sequence and structure data can help us to uncover these mechanisms.

